# Regulation of Cholesterol Metabolism by Bioactive Components of Soy Proteins: Novel Translational Evidence

**DOI:** 10.3390/ijms22010227

**Published:** 2020-12-28

**Authors:** Giusy Rita Caponio, David Q.-H. Wang, Agostino Di Ciaula, Maria De Angelis, Piero Portincasa

**Affiliations:** 1 Department of Soil, Plant and Food Sciences, University of Bari Aldo Moro, via Amendola 165/a, 70126 Bari, Italy; giusy.caponio@uniba.it; 2 Division of Internal Medicine Clinica Medica “A. Murri”, Department of Biomedical Sciences and Human Oncology, University of Bari Aldo Moro, 70124 Bari, Italy; agostinodiciaula@tiscali.it; 3 Department of Medicine and Genetics, Division of Gastroenterology and Liver Diseases, Marion Bessin Liver Research Center, Einstein-Mount Sinai Diabetes Research Center, Albert Einstein College of Medicine, Bronx, NY 10461, USA; david.wang@einsteinmed.org

**Keywords:** cholesterol, soybean, proteins, health, cardiovascular diseases

## Abstract

Hypercholesterolemia represents one key pathophysiological factor predisposing to increasing risk of developing cardiovascular disease worldwide. Controlling plasma cholesterol levels and other metabolic risk factors is of paramount importance to prevent the overall burden of disease emerging from cardiovascular-disease-related morbidity and mortality. Dietary cholesterol undergoes micellization and absorption in the small intestine, transport via blood, and uptake in the liver. An important amount of cholesterol originates from hepatic synthesis, and is secreted by the liver into bile together with bile acids (BA) and phospholipids, with all forming micelles and vesicles. In clinical medicine, dietary recommendations play a key role together with pharmacological interventions to counteract the adverse effects of chronic hypercholesterolemia. Bioactive compounds may also be part of initial dietary plans. Specifically, soybean contains proteins and peptides with biological activity on plasma cholesterol levels and this property makes soy proteins a functional food. Here, we discuss how soy proteins modulate lipid metabolism and reduce plasma cholesterol concentrations in humans, with potential outcomes in improving metabolic- and dyslipidemia-related conditions.

## 1. Introduction

Legumes are among the vegetables with the highest protein content. As food, legumes represent the basis of cell metabolism in every living organism. Unlike food of animal origin, legumes have a very low unsaturated fat content (2–4%) and because they are plant foods, they are cholesterol free as well [1,2]. Legumes contain a high quantity of starch with a fair amount of dietary fibers, good source of energy, and are free of gluten. In addition, legumes contain the highest concentrations of vitamins (group B), iron, calcium, and potassium among all other vegetables. Among legumes, soybean—an annual leguminous plant that was first cultivated in China five thousand years ago [3]—is of particular interest due to the potential effects on cholesterol homeostasis in the body. Soybean can partly replace animal proteins, since it contains amino acids that are essential to human nutrition. Consumption of soybean has increased over the years, due to the combination of organoleptic properties, and has shown beneficial effects on health, including potential protective actions on the cardiovascular system and lipid metabolism. This review will discuss the mechanisms of action of soy proteins and peptides on cholesterol metabolism and potential beneficial reflections on human health.

## 2. The Burden of Cardiovascular Diseases (CVD) as Components of the Metabolic Syndrome

CVD is the leading cause of morbidity and mortality worldwide. According to the World Health Organization, CVD is the first cause of death globally, with an estimated 17.9 million deaths each year and nearly 23.6 million deaths by 2030 [4,5].

As reported by the American Heart Association, four out of five CVD-related deaths are due to heart attacks and strokes, and one third of these deaths occur prematurely in people under 70 years of age [6]. About 48% of subjects above 20 years of age have CVD in the United States [7]. The reported prevalence of CVD increases with age in both sexes. The term CVD encompasses four main groups of disorders of the heart and blood vessels, which include (i) chronic heart disease, manifesting as myocardial infarction, angina pectoris, heart failure, and coronary death; (ii) cerebrovascular disease, manifesting as stroke and transient ischemic attack; (iii) peripheral artery disease, manifesting as intermittent claudication; and (iv) aortic atherosclerosis and thoracic or abdominal aortic aneurysm. Identification of subjects at increased risk of CVD allows appropriate management and can prevent premature deaths. Nevertheless, millions of people worldwide struggle to control the risk factors for CVD, while many others remain unaware that they are at high risk. A large number of heart attacks and strokes are preventable by controlling major risk factors through lifestyle interventions and drug treatment, if necessary. The risk factors for CVD include behavioral factors, such as tobacco use, and unhealthy diets rich in fat, carbohydrates, and salt, as well as excessive consumption of alcohol, and sedentary life. Additional risk factors are arterial hypertension, and dyslipidemia, namely, increased total cholesterol, low-density lipoprotein-cholesterol (LDL-C), low high-density lipoprotein-cholesterol (HDL-C), and triglyceride concentrations in plasma, as well as insulin resistance and diabetes mellitus, overweight, and obesity, with all highly likely being components of the metabolic syndrome. Social determinants linked to the increased prevalence of CVD are ageing, income, and urbanization. The characteristics of CVD encompass a spectrum of pathogenic factors, namely, accumulation of excess cholesterol, inflammatory response, cell death, and fibrosis in the arterial wall.

The evaluation of dyslipidemia, including elevated total cholesterol and LDL-C, and familial hypercholesterolemia plays a key role in CVD assessment [8]. Cholesterol is the major sterol in humans and is the key component of mammalian cell membranes. In the human plasma, cholesterol is present as unesterified (“free”) and esterified forms, with both being approximately 30 and 70%, respectively. By contrast, biliary cholesterol is 95% free cholesterol. The concentrations of plasma cholesterol in humans range from 120 to 200 mg/dL as a component of LDL-C, HDL-C, and VLDL. Biliary cholesterol concentrations are even higher, i.e., about 390 mg/dL since the two cholesterol carriers, i.e., bile acids and phospholipids, form micelles and vesicles, which further increase cholesterol solubilization in bile [9]. Several pathways contribute to the net flow of cholesterol through the major tissue compartments of the human body, a process keeping the cholesterol pool essentially constant. Two sources provide cholesterol to the body, i.e., intestinal absorption of cholesterol from dietary and biliary sources, and cholesterol biosynthesis in various tissues. Two major pathways govern the excretion of cholesterol from the body, i.e., bile and the gastrointestinal tract. In addition, cholesterol is converted to BA and steroid hormones. In the steady state, the total input of cholesterol into the human body is equal to the total output. Children and growing animals, however, have a greater input of cholesterol into the body than the output, and the accumulation of cholesterol contributes to body weight gain. The available dietary and biliary cholesterol to the body varies largely in different individuals, but the total amount of cholesterol from the small intestine to the body depends mainly on the absorption efficiency of intestinal cholesterol and the amount of cholesterol consumed daily [10,11,12,13,14,15,16,17,18,19,20,21].

**Figure 1:**
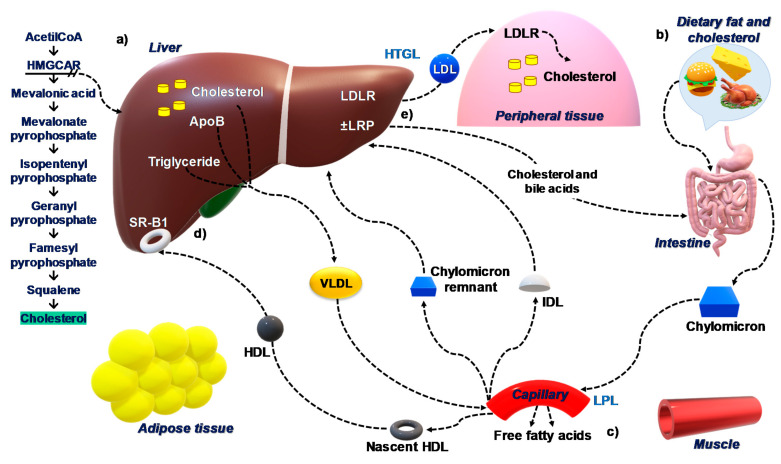
Cholesterol homeostasis in the body. (**a**) The rate of cholesterol synthesis by the liver is highly dependent on the cellular level of cholesterol and the absorption rate of the small intestine. This feedback regulation is determined predominantly by sterol regulatory element-binding protein-2 (SREBP-2). Subsequently, 3-hydroxy-3-methylglutaryl coenzyme A reductase (HMGCR) catalyzes the formation of mevalonate, the rate-limiting step in cholesterol biosynthesis. (**b**) Cholesterol and triglycerides from diets are absorbed in the small intestine and are incorporated into chylomicrons. (**c**) In the plasma, lipoprotein lipase (LPL) acts on chylomicrons to generate fatty acids and chylomicron remnants that are taken up by the liver. The nascent HDL is generated by lipolysis mediated by LPL of lipoproteins rich in triglycerides including VLDL. (**d**) The concentration of free cholesterol is ”sensed” by SREBP-2 that activates cholesterol synthesis enzyme, HMGCR, when cholesterol content in the hepatocytes is reduced by the import of lipoproteins or is increased by the conversion to bile acids. In addition, SREBP-2 also activates the synthesis of LDL receptors (LDLR), accelerating the uptake of cholesterol from LDL and then promoting the storage of cholesterol in the liver. (**e**) Conversely, when cholesterol content in the hepatocytes is increased, SREBP-2 is inhibited, leading to LDLR downregulation and inactivation of cholesterol synthesis. Abbreviations: LDLR, low-density lipoprotein receptor; HMGCR, 3-hydroxy-3-methylglutaryl coenzyme A reductase; VLDL, very low-density lipoprotein; HDL-C, low high-density lipoprotein-cholesterol; LPL, lipoprotein lipase; SREBP-2, sterol regulatory element-binding protein-2.

The absorption of biliary cholesterol represents about two thirds of the total daily amount of cholesterol originating from the intestine [22]. The overall picture of cholesterol homeostasis in the body is depicted in Figure 1.

**Figure 2:**
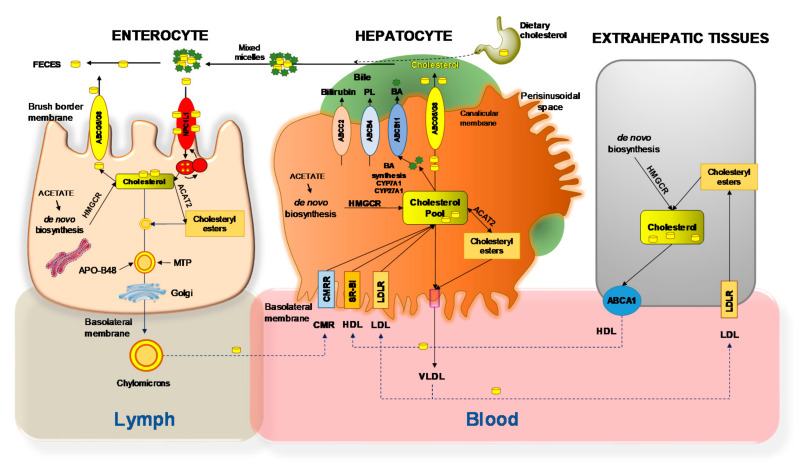
Net cholesterol flow through the body. In the small intestinal lumen, micellar solubilization facilitates the diffusion of sterols through enterocyte. Mixed micelles (Bile acids (BA) and cholesterol) increase the uptake of cholesterol via the Niemann–Pick C1 like 1 (NPC1L1) protein, a sterol influx transporter. The transporter ABCG5/G8 (brush border membrane) promotes active efflux of cholesterol from the enterocyte back into the intestinal lumen for fecal excretion. The combined regulation of NPC1L1 and ABCG5/G8 modulates the amount of cholesterol reaching the lymph from the intestine. Cholesterol (either absorbed or newly synthesized molecules from acetate by 3-hydroxy-3-methylglutaryl-CoA reductase [HMGCoAR]) are esterified to fatty acids by acyl-CoA:cholesterol acyltransferase isoform 2 (ACAT2), to form cholesteryl esters. This pool of lipids contributes to the assembly of chylomicrons, which are enriched with the apolipoprotein B-48 (apoB-48) and require microsomal triglyceride transfer protein (MTTP). The core of chylomicrons secreted in lymph contains triglycerides and cholesteryl esters. The surface contains phospholipids, unesterified cholesterol, and apolipoproteins such as apoB-48, apoA-I, and apoA-IV [24,25]. The hepatic uptake of cholesterol involves the scavenger receptor class B type I (SR-BI) for high-density lipoprotein (HDL), the low-density lipoprotein (LDL) receptor (LDLR) for LDL, and the chylomicron remnant receptor (CMRR) for chylomicron remnants (CMR). Biosynthesis of hepatic cholesterol (CH) from acetate requires the rate-limiting enzyme 3-hydroxy-3-methylglutaryl-coenzyme A reductase (HMGCR). Most of the cholesterol undergoes the synthesis of BA involving two rate-limiting enzymes in the classical pathway (cholesterol 7a-hydroxylase, CYP7A1) and the alternative pathway (sterol 27-hydroxylase, CYP27A1). Some cholesterol is esterified by acyl-coenzyme A: cholesterol acyltransferase isoform 2 (ACAT2). This step provides lipid droplets serving for storage within the hepatocytes. Some of the cholesterol is arranged with the very-low-density lipoprotein (VLDL), secreted into blood. Cholesterol secretion across the canalicular membrane of hepatocytes into the bile canaliculus requires the specific ATP-binding cassette transporter ABCG5/G8. This process is paralleled by secretion of the two other lipids acting as cholesterol transporters in bile, i.e., BA via ABCB11, and phospholipids (PL) via ABCB4. In extrahepatic tissues VLDL delivers cholesterol across LDL and LDLR, while cholesterol is excreted via the transporter ABCA1 and formation of HDL. Abbreviations: CYP7A1, cholesterol 7-α; CYP27A1, sterol 27-hydroxylase; RER, rough endoplasmic reticulum. Adapted from D. Q-H. Wang et al. [26,27,28].

The detailed processes involving the net flow of cholesterol through the major human and animal tissue compartments of the body is shown in Figure 2. At the enterocyte level, the Niemann–Pick C1-like 1 (NPC1L1) protein is expressed at the apical membrane, and plays a critical role in the ezetimibe-sensitive cholesterol absorption pathway [9,23].

The absorbed cholesterol from the small intestine can regulate hepatic cholesterol synthesis, depending on the amount of daily food intake, through a negative feedback regulatory mechanism. In particular, the regulatory mechanisms on cholesterol metabolism appear to finely adjust the rate of cholesterol biosynthesis in the body and the rate of cholesterol excretion from the body. This pathway accommodates the varying amounts of cholesterol absorbed by the small intestine any time with little net accumulation of excess cholesterol in the body, still meeting the metabolic needs of cells. If the fine regulatory mechanisms are failed, there is a significant increase in plasma cholesterol concentration and/or hepatic cholesterol secretion in humans [19,29,30,31,32].

In the cardiovascular system, abnormal metabolism of cholesterol leads to the accumulation of excess cholesteryl esters in the arterial wall, and this is a key step in atherosclerosis and cardiovascular disease [33,34,35,36,37,38,39]. In this pathophysiologically relevant scenario, dietary fat is greatly involved in several processes in fatty acid metabolism [28,40]. On one hand, the diet is a source of saturated fatty acids, which could increase plasma LDL-C levels and atherosclerotic risk. On the other hand, a “healthy” diet is rich in monounsaturated and polyunsaturated fatty acids and increases the ratio of unsaturated/saturated fatty acids. The unsaturated fatty acids are often provided from olive oil, nuts, fruits, vegetables, and legumes. In addition, the Mediterranean diet is rich in dietary fibers, which may reduce intestinal absorption of lipids and carbohydrates [41]. A diet rich in essential omega 6 and omega 3 fatty acids may add beneficial effects against hypertension, hypercholesterolemia, hypertriglyceridemia, thrombosis, and type 2 diabetes mellitus [42].

Moreover, certain foods are a unique source of “active ingredients”, such as antioxidants, vitamins, minerals, polyunsaturated fatty acids, which may make humans healthier. Polyphenols, for example, have anti-inflammatory actions and promote health in certain chronic diseases [43,44]. Thus, diet as a component of healthy lifestyles becomes a true additional therapeutic agent involved in prevention and treatment of metabolic disorders [45,46]. Some evidence suggests that the Mediterranean diet may reduce the risk of metabolic disorders and improve the quality of life [47,48,49].

Table 1 lists the main beneficial nutrients in the Mediterranean diet. If other diets are enriched with nutrients typical of the Mediterranean diet, they can also be considered “healthy” (“prudent”) [50].

## 3. Soy Proteins

**Figure 3:**
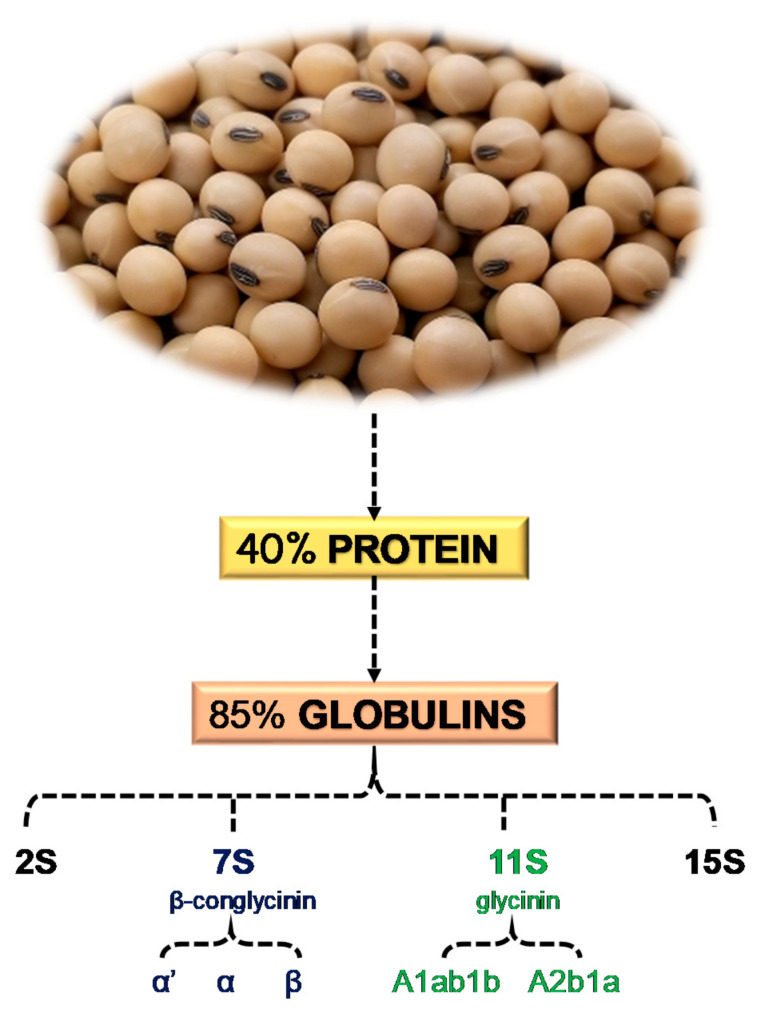
Summary of the main components of soybean proteins [55,56,57,58,61].

Several studies investigated the health benefits of soybeans [51,52,53], as also certified by the Food and Drug Administration [54]. Soybeans contain approximately 40% protein and 20% oil on an average dry matter base [55] (Figure 3). About 85% of proteins are globulins [56], which are divided into four categories: 2S, 7S, 11S, and 15S, based on sedimentation rate [57]. The two principal soy proteins are β-conglycinin (7S globulin) and glycinin (11S globulin), and their hydrolysis with protease generates biologically active peptides. Beta-conglycinin has three subunits (α’, α, and β), and is involved in the hypolipidemic action of soybeans [58]. Lovati et al. showed that 7S globulin and peptides generated by its hydrolysis play a role in lipid metabolism by regulating LDLR activity [59]. Matoba et al. performed in vitro experiments to study the digestion of subunit 7S α’ with endoproteases and their capacity of entering the bloodstream to carry out anti-hypertensive activity [60]. Glycinin and β-conglycinin have sequestering action on bile acids, reduce intestinal absorption of cholesterol, and promote a plasma cholesterol-lowering effect. The subunits involved are A1ab1b and A2b1a of soy glycinin [61]. In addition, glycinin possesses some biologic activities, such as angiotensin-converting enzyme inhibition, antithrombotic activity, and antioxidant properties [62].

## 4. Effects of Soy Proteins and Peptides on Lipid Metabolism

Soy proteins and peptides exert an interesting cholesterol-lowering activity based on cell culture experiments [63]. Lammi et al. examined the activity of three peptides from soy glycinin hydrolysis—IAVPGEVA, IAVPTGVA, and LPYP. The cholesterol-lowering effect of three peptides may depend on their ability to increase LDLR activity, which can internalize the extracellular LDL-C in cultured HepG2 cells. Three peptides act in vitro as competitive HMGCR inhibitors for regulating cholesterol biosynthesis. This, in turn, results in a reduction in intracellular cholesterol synthesis, leading to the activation of SREBP-2 and increasing uptake of LDL by HepG2 cells. In addition, these peptides may reduce the production of cholesterol levels through the activation of the AMPK pathway—with an increase in phosphorylation at Thr173. This step, in turn, leads to an inactivation of its target substrate HMGCR via phosphorylation at serine 872. Moreover, it may promote the activation of ERK1/2 which could lead to a stabilization of mRNA levels of LDLR for increasing levels of LDLR protein in the plasma membrane [63]. IAVPGEVA, obtained from the pepsin hydrolysate of glycinin may work with bile acids to reduce cholesterol content in plasma [64]. This peptide has an in vitro effect to inhibit HMGCR activity by 75%.

**Figure 4:**
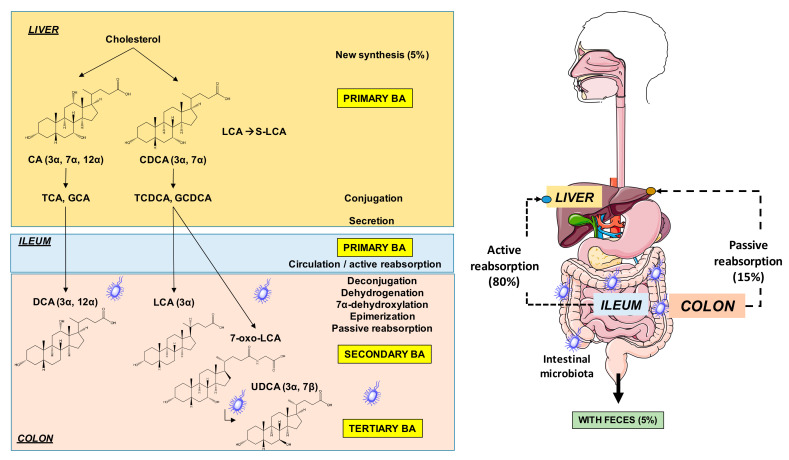
Sites of synthesis and metabolism of primary, secondary, and tertiary bile acids in humans. Abbreviations: CA, cholic acid; CDCA, chenodeoxycholic acid; DCA, deoxycholic acid; LCA, lithocholic acid; UDCA, ursodeoxycholic acid.

The effect of soy proteins might also involve the gut microbiota and bile acid homeostasis [65,66]. It has been found that the peptide IAVPGEVA significantly binds primary bile acid, cholic acid (CA), and secondary bile acid, deoxycholic acid (DCA) converted from CA by colonic microbiota [65,66] (Figure 4). The hypocholesterolemic effect of pepsin hydrolysate likely involves the binding of digested fragments of dietary fiber to bile acids, reducing bile acid reabsorption in the small and large intestines and increasing liver synthesis of bile acids from their precursor, cholesterol [67]. Therefore, the peptide IAVPGEVA exerts its hypocholesterolemic action by seizing bile acids.

β-Conglycinin is another soy protein that contains peptides which are competitive HMGCR inhibitors and, therefore, display a statin-like mechanism. A study was performed with YVVNPDNDEN and YVVNPDNNEN—two peptides from soybean β-conglycinin—to investigate their interactions with the catalytic site of HMGCR using molecular modeling tools and to characterize the molecular mechanism through which they potentially mediate a hypocholesterolemic effect in HepG2 cells. Compared to the untreated sample, peptides upregulated the mature SREBP-2 protein level (by 134.0 ± 10.5% with YVVNPDNDEN and by 158.0 ± 9.2% with YVVNPDNNEN), increased LDLR protein levels (by 152.0 ± 20.0% with YVVNPDNDEN and by 164.0 ± 17.9% with YVVNPDNNEN), and increased the production of HMGCR protein (by 171 ± 29.9% with YVVNPDNDEN and by 170 ± 50.0% with YVVNPDNNEN). Thus, peptides by increasing intracellular levels of LDLR protein through activation of SREBP-2, allow HepG2 cells to uptake LDL-C proportionally to the molecular increase in intracellular levels of LDLR protein [68]. In addition, the activation of LDLR has been found in (HepG2) cells challenged with soybean β-conglycinin [69].

Another study investigated whether the two subunits, α and α’, of β-conglycinin are responsible for the biochemical effect [70]. The effect of the commercially available isoflavone-free soy protein concentrate (Croksoy) on LDLR of HepG2 cells is compared with a variant of soy cultivar (Keburi mutant) that is deficient in the α’ subunit of β-Conglycinin. The Croksoy and not the Keburi variant (α’ free) induces upregulation of LDLR (73% increase in uptake and degradation of LDL at 0.75 g/L concentration), implying that the subunit α’ may be responsible for the upregulation of LDLR.

Lovati et al. [59] studied both the activation of LDLR exposed to α’ + α subunits of soy globulin 7S and Croksoy^R^70—a commercial isoflavone-free soybean concentrate—and their ability to interfere in the secretion of apolipoprotein (apo) B in the medium and sterol biosynthesis. Cells exposed to Croksoy^R^70 enzyme digestion products showed a more marked upregulation of LDLR vs. controls, compared with vs. Hep G2 cells incubated with undigested Croksoy^R^70. Moreover, 7S globulin inhibited apo B secretion, and 14C-acetate incorporation when tested in Hep G2 cells at a concentration of 1.0 g/L.

FVVNATSN is another peptide of β-Conglycinin that increases LDLR transcription [71]. Among the various peptides tested in vitro, FVVNATSN at a concentration of 100 µm had the strongest activity in increasing LDLR transcription in hepatocytes (+248.8%, compared to 100% of untreated control).

Lunasin—small subunit of a 2S albumin protein in soybean [72]—is an active component responsible for decreased LDL-C. The mechanism might involve the lunasin-mediated inhibition of H3-Lysine 14 acetylation by PCAF histone acetylase enzyme, reduced expression of HMGCoA reductase, and increased LDLR expression. The effect of soy proteins and peptides on lipid metabolism is summarized in Table 2.

**Figure 5:**
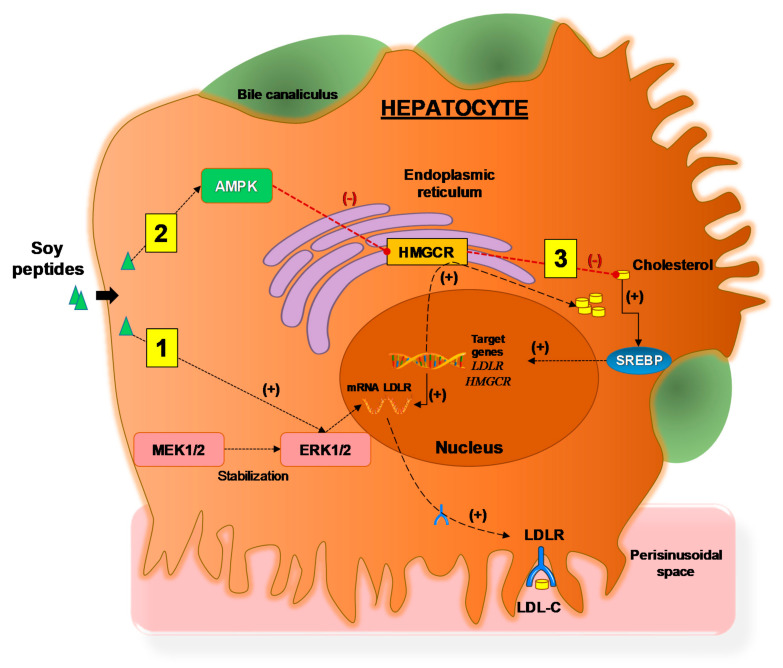
The mechanism of action of soy peptides in HepG2 cells, adapted from Lammi et al., 2015 [63]. Three distinct pathways lead to increased LDLR activity, which can bind and transport extracellular LDL into liver cells with final hypocholesterolemic effects. (1) Soy peptides, which enter the pathway that stabilizes mRNA levels contributing to the increased levels of LDLR protein in the plasma membrane and increased LDL-C uptake. (2) Soy peptides activate AMPK which, by phosphorylation inactivates the target substrate HMGCR, the key enzyme involved in cholesterol synthesis. This step is associated with decreased intracellular level of cholesterol. (3) Decreased intracellular cholesterol levels activate the transcription factor SREBP-2 which, in turn, activates the transcription of genes *LDLR* and *HMGCR* resulting in increased levels of proteins LDLR and HMGCR, respectively. Abbreviations: AMPK, adenosine monophospate-activated protein kinase; ERK1/2, extracellular signal-regulated protein kinases 1 and 2; HMGCR, 3-hydroxy-3-methylglutaryl coenzyme A reductase; LDL-C, low-density lipoprotein-cholesterol; LDLR, low-density lipoprotein receptor; MEK1/2, mitogen-activated protein kinases 1 and 2; SREBP, Sterol regulatory element-binding protein. (+), increased effect; (−), decreased effect.

The intracellular effects of soy peptides in hepatocyte are depicted in Figure 5.

## 5. Effect of Soy Proteins on Lipid Metabolism—Clinical Evidence

The potential cholesterol-lowering effects of soy proteins are of interest in clinical medicine. The effect of soy proteins on lipid metabolism has been investigated in different populations, namely, hypercholesterolemic patients, type 2 diabetes, overweight or obese patients, and healthy individuals.

The first human study on the lipid-lowering effect of soy proteins was performed in 1967 [73], and showed that in hypercholesterolemic men, plasma cholesterol levels were decreased from 295 to 172 mg/mL after 4 weeks on a diet with vegetable proteins, including mainly purified soy proteins. Other studies compared the effect of diet containing soy proteins with that of the diet containing animal proteins for 4 weeks. Soy proteins reduced total cholesterol by 15.9% and LDL-C by 16.4%, and the mechanism is likely involved in the uptake of LDL by mononuclear cells, which is increased by 16-fold compared to the basal activity and by 8-fold compared to the standard diet with low lipids and animal proteins [74]. In children, soy protein consumption significantly reduces the concentrations of triglycerides and VLDL-C, and increases the concentrations of HDL-C and HDL_3_-C, but does not influence either plasma total cholesterol, LDL-C, or apo concentrations [75]. In another study, soy protein intake for 14 days also increased fecal sterol excretion compared to the casein intake, and reduced plasma cholesterol levels [76].

Sagara et al. compared diets containing at least 20 g soy proteins and 80 mg isoflavones with placebo diets in middle-aged hypercholesterolemic men. Soy protein diets for 5 weeks are associated with a significant reduction from baseline in both systolic and diastolic blood pressure, total- and non-HDL-C, and an increase in HDL-C in both the soy proteins and the placebo groups [77].

Wang et al. compared the effect and mechanisms of soy proteins with those of animal proteins from meat and dairy because they have different types of fatty acids and isoflavones [78]. The study showed that soy protein intake is better than animal protein intake to induce a significant reduction (−13.3%) of the fatty acid fractional synthetic rate, and increase (+7.6%) in free cholesterol fractional synthetic rate. In addition, isoflavones reduce total cholesterol concentrations by 3.1% and do not have an effect on LDL-C, HDL-C, or triglyceride concentrations, as well as free cholesterol fractional synthetic rate and fatty acid fractional synthetic rate.

Two studies also confirmed that daily intake of soy proteins at 25 g for 4 weeks [79] and at 30 g for 12 weeks [80] reduces plasma total cholesterol, LDL-C, insulin, and apoB concentrations. Moreover, soy proteins may be specifically associated with decreased LDL-C and apoB levels, as well as the ratio of plasma LDL-C to HDL-C. There is no change in HDL-C, apo A-I, lipoprotein (a), and triacylglycerol concentrations [81,82]. Consuming 40 g soy proteins that contain 1.39 to 2.25 mg isoflavones/g protein for six months reduces non-HDL-C and increases LDLR mRNA in mononuclear cells and HLD-C levels compared to those of casein and non-fat dry milk in postmenopausal women [83,84]. In addition, eating 28 g soy proteins daily—with two different amounts of isoflavones, 65 mg or 4 mg—reduces total cholesterol, LDL-C, and sex hormone binding globulin concentrations in postmenopausal women. However, there is no difference between the two doses of isoflavones, suggesting that lipid lowering effect may be independent from isoflavones [85].

A recent meta-analysis discussed 43 controlled clinical trials, and showed that intake of soy proteins at 25g/day for 6 weeks significantly decreases plasma LDL-C concentrations by 4.76 mg/dL and total cholesterol concentrations by 6.41 mg/dL in hypercholesterolemic men and women [86]. Plasma LDL-C, apoB100, triglyceride, and homocysteine concentrations, as well as LDL/HDL ratio are reduced also in type 2 diabetic individuals and overweight or obese individuals after 6 and 12 weeks of consumption of soy proteins [87,88].

For healthy subjects, daily consumption of soy proteins at 25 g/day for 9 weeks [89], or at 20 g/day for 18 weeks [90], or at 64 or 128 mg/day for 3 menstrual cycles plus 9 days with a 2–3 weeks washout between diet periods [91], reduces total cholesterol and LDL-C levels and does not influence plasma triglyceride and HDL-C concentrations.

There is a significant reduction in total cholesterol levels in pre-menopausal healthy women supplemented with soy milk at 408 g/day [92,93]. In addition, there is a significant reduction in total cholesterol levels and LDL-C/HDL-C ratio in post-menopausal healthy women supplemented with soy foods plus isoflavones at 40–60 mg/day [92,93].

When men and postmenopausal women are given 40 g soy proteins plus a much higher dose of isoflavones at 118 mg/day for 3 months, triglyceride and low- to high-density lipoprotein ratio are reduced, without change in total cholesterol, LDL-C, and HDL-C concentrations [94].

Rosell et al. showed that intake of soy proteins is inversely associated with total cholesterol and LDL-C concentrations and with the ratio of total cholesterol to HDL-C, but not HDL-C, concentrations in pre- and post-menopausal women. Soy protein consumption at 6 g/day leads to 12.4% lower average plasma LDL-C concentrations compared to those at 0.5 g/day in women [95].

Similar findings have been found in healthy men and women eating soy proteins for one year [96]. Consumption of soy proteins at <8 g/day also reduces total cholesterol concentrations in both men and women.

Mixture of non-dairy cheese cream, containing 75% fermented soybean extract, causes a slight increase in plasma lipid profile during oral fat load compared to a dairy cheese cream. Notably, fermented soybean extract decreased total cholesterol, triglyceride, and LDL-C, but not HDL-C, concentrations in healthy men and women [97]. However, the hypolipidemic benefits of soy proteins in other studies using 40 g/day of soy proteins in 135 healthy men or women were insignificant [98]. Consumption of soy proteins has positive effects on some indices of bone metabolism and has little effect on plasma total cholesterol, LDL-C, and HDL-C concentrations in normolipidemic and mildly hyperlipidemic men and women. This could probably be associated with the wide age range of participants that were selected for the study (i.e., 27–87 years old) [98].

In healthy men and women, a treatment with lunasin enriched soybean extract (335 mg) was not able to decrease total cholesterol and LDL-C levels, nor triglycerides and glucose levels, insulin resistance, blood pressure, BMI, or waist circumference [99]. The main clinical controlled trials involving intake of soy products are reported in Table 3.

## 6. Discussion

The impact of cardiovascular disease is enormous in modern societies. In addition to the standard pharmacological approaches, lifestyles also play an important role in both primary and secondary prevention. Whereas unhealthy diets may contribute to the pathogenesis of these diseases [100], some types of foods and nutrients can bring beneficial effects because of their healthy dietary patterns [101,102].

As a general recommendation, dietary proteins should represent 10–35% of total caloric intake. As part of the healthy diet, individuals should eat a variety of protein-rich foods, i.e., lean meat, poultry, fish, eggs, unsalted nuts and seeds, beans, and peas.

Soy products represent another source of healthy proteins, while patients should avoid those protein sources with unhealthy fat. Some evidence has suggested that soy components might have a positive effect on lowering plasma cholesterol concentrations, with some mechanisms being clarified from in vitro experiments.

In addition, few human studies have been carried out at a translational level. A recent paper identified 114 meta-analyses and systematic reviews with 43 unique outcomes [103]. Soy proteins and isoflavone consumption may have more beneficial actions than harmful effects on a variety of health outcomes, i.e., cardiovascular disease, and cancers, as well as gynecological, metabolic, musculoskeletal, endocrine, neurological, and renal outcomes. The results of this paper support the concept that soy protein intake could work as part of a healthy diet for humans.

In a recent study in Japanese adults, habitual intake of soy food or isoflavones was not associated with 5-year changes in plasma lipid and HbA1c concentrations. However, there was a negative association between intake of fermented soy (“natto”) and changes in plasma lipid concentrations in overweight or obese subjects [104]. Overall, these findings require more randomized controlled trials.

Gut BA undergo key biotransformation due to resident microbiota [105] Since soy proteins can interact with gut BA, further studies need to address the relationships between BA, gut microbiota, and soy products 

**Figure 6:**
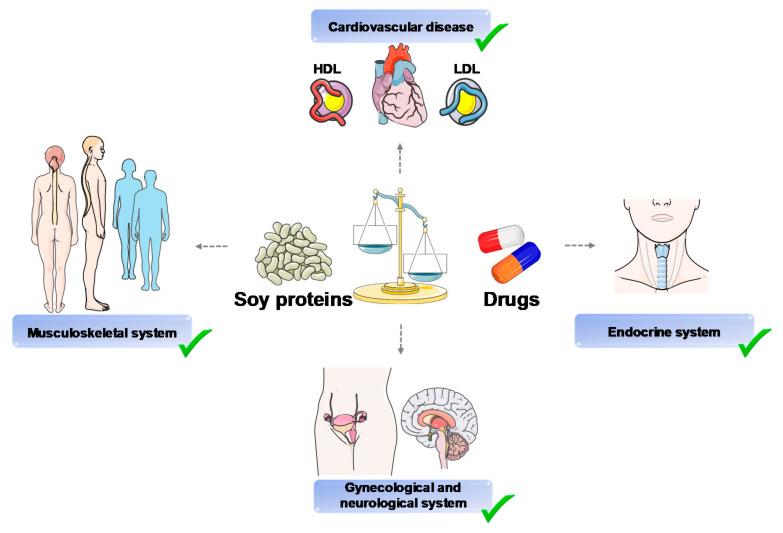
Summary of main healthy benefits of soy proteins. Abbreviations: HDL, high-density lipoprotein; LDL, low-density lipoprotein. Images freely available from Servier Medical Art.

As shown in Figure 6, some nutritional advantages could be obtained by replacing many drugs with soy foods, specifically soy proteins. Therefore, the possible use of soybean in functional food design appears promising in terms of both primary and secondary prevention.

## 7. Conclusions

Soybean is an extraordinary source of high-quality plant protein which displays functional properties. Soy proteins play a role in metabolic disease; are involved in serum cholesterol reduction and decrease in blood pressure; and have effects on menopause, diabetes, and insulin resistance. We highlighted the role of soy proteins and soy products as a functional food beneficial on human health.

## Figures and Tables

**Table 1:** The main beneficial components in the Mediterranean diet.

Food	Recommended Doses	Components
Extra-virgin olive oil	50 g/day	Polyphenols
Tree nuts and peanuts	30 g/day	Essential omega 6 and omega 3 fatty acids
Fresh fruits/vegetables	≥3 servings/day	Flavonols, vitamins
Fish, seafood	≥3 servings/week	Essential omega 6 and omega 3 fatty acids
Legumes	≥3 servings/week	Fibers, proteins

**Table 2:** Effect of soy proteins and peptides on lipid metabolism.

Soy Protein	Peptides	Doses	Experimental Model	Effects/Bioactivity	References
Glycinin (11S)	IAVPGEVA, IAVPTGVA, LPYP	25, 50, 100, 150, 200, 250, 300, 400, 500, 650, 1000 µM	HepG2 cell line	Increased LDLR activity Inhibition of HMGCR Activation of the AMPK pathway and ERK1/2	[63]
	IAVPGEVA	-	Microsomes from rat liver	Inhibition of HMGCR Seizure of bile acids	[64]
β-Conglycinin (7S)	YVVNPDNDEN, YVVNPDNNEN	350 and 500 μM	HepG2 cell line	Inhibition of HMGCR Upregulation of the mature SREBP-2 protein level Increased the level of LDLR protein	[68]
	α, α′ subunits	0.25, 0.50, 0.75 g/L	HepG2 cells line	Upregulation of the LDLR	[70]
	α, α′ and β subunits	10^−4^ mol/L	HepG2 cells line	Upregulation of the LDLR	[59]
	FVVNATSN	100 µM	Human hepatocytes (Hep T9A4)	Influenced LDLR transcription	[71]
2S albumin	Lunasin	-	HepG2 cells line	Reduced expression of HMGCR	[72]

Abbreviations: AMPK, AMP-activated protein kinase; LDLR, low-density lipoprotein receptor; HMGCR, 3-hydroxy-3-methylglutaryl coenzyme A reductase; ERK1/2, extracellular signal-regulated protein kinases 1 and 2; SREBP-2, Sterol regulatory element-binding protein-2.

**Table 3:** Main clinical controlled trials involving intake of soy products.

Authors	Sample Size	Gender (Age)	Format, Dose	Duration of Study	Main Findings
*Hypercholesterolemia*					
Hodges et al., 1967 [73]	N = 6	Men (33–46 years)	n.a.	4 weeks	Reduction in the average plasma cholesterol levels by more than 100 mg/mL.
Lovati et al., 1987 [74]	N = 12	5 men and 7 women (26–64 years)	Soy proteins (20% calories)	4 weeks	Reduction in total cholesterol by 15.9% and LDL-C by 16.4%. Degradation of LDL-C by mononuclear cells after soybean diet (increased 16-fold vs. the basal activity and 8-fold compared with the standard low lipid diet with animal proteins).
Laurin et al., 1991 [75]	N = 55	Children (6–12 years)	Soy beverage (250 mL)	4 weeks	No changes in either plasma total cholesterol, LDL-C, or apo concentrations. Reduction in triglyceride and VLDL concentrations. Increased HDL-C and HDL_3_-C concentrations.
Wang et al., 1995 [76]	n.a.	Females (median age 19 years)	Soy proteins, purified (4 or 8% of total energy)	14 days	Decreased LDL-C, increased HDL-C and increased fecal steroid excretion.
Baum et al., 1998 [84]	N = 66	Postmenopausal women (39–83 years)	Soy proteins (40 g/day containing 1.39 or 2.25 mg isoflavones/g protein)	6 months	Decreased non-HDL-C levels. Unchanged plasma total cholesterol levels. Increased HDL-C levels and decreased ratio total cholesterol and HDL-C in both groups compared with control. Increased LDLR messenger RNA concentrations in mononuclear cells.
Potter et al., 1998 [83]	N = 66	Postmenopausal women (39–83 years)	Soy proteins at 40 g/day (containing 1.39 or 2.25 mg isoflavones/g protein)	6 months	Reduction in non-HDL-C levels. Increased HDL-C levels. Increased mononuclear cell LDLR mRNA.
Wong et al., 1998 [81]	N = 13	Men (20–50 years)	Soy beverage (≥75% of the total protein content of diet)	5 weeks	Decreased LDL-C and LDL-C/HDL-C ratio.
Mackey et al., 2000 [85]	N = 54	Postmenopausal women (median age 56 years)	Soy proteins (28 g with 65 mg or 4 mg isoflavones)	12 weeks	Reduction in TC, LDL-C, sex hormone binding globulin (SHBG), and luteinizing hormone (LH). No significant differences between treatment groups. Cholesterol-lowering effect in both women and men independent of isoflavones.
Teixeira et al., 2000 [82]	N = 81	Men (23–74 years)	Soy proteins (20 g/day)	6 weeks	Reduced non-HDL, total cholesterol and apoB. No change in HDL-C, apoA-I, lipoprotein(a), and triglyceride concentrations.
Maki et al., 2000 [80]	N = 30	13 men and 17 women (18-79 years)	Soy proteins (25 g/day)	4 weeks	Reduced atherogenic lipoproteins, as indicated by changes in total cholesterol (27.4 and 23.6%), LDL-C (210.9 and 25.9%), non-HDL-C (210.8 and 23.9%), and apoB (29.7 and 22.4%), respectively.
Blanco Mejia, 2019 [86]	N = 2607	37% men and 63% women (median age 54.9 years)	Soy proteins (25 g/day)	6 weeks	Decreased LDL-C by 4.76 mg/dL and total cholesterol by 6.41 mg/dL.
*Type 2 diabetes*					
Hermansen et al., 2001 [87]	N = 20	14 men and 6 women (median age 63.6 years)	Soy proteins (50 g/day) plus high levels (minimum 165 mg/day) of isoflavones	6 weeks	Reduction in LDL-C, LDL/HDL ratio, apoB100, triglyceride, and homocysteine concentrations.
*Overweight/obesity*					
Anderson et al., 2005 [88]	N = 51	4 men and 47 women (18–65 years)	Five soy-based meal replacements (Soy MR)/day	12 weeks	Decreased plasma total cholesterol, LDL-C, and triglyceride concentrations.
*Healthy subjects*					
Nagata et al., 1998 [96]	N = 4838	1242 men and 3596 women (median age 58.38 years)	Soy proteins (<8 g/day)	1 year	Decreased total cholesterol concentrations with an increasing intake of soy products in men and women after controlling for age, smoking status and intake of total energy, total proteins and total fat.
Crouse et al., 1999 [89]	N = 156	94 men and 62 pre and postmenopausal women (20–70 years)	Isolated soy proteins (25 g) plus isoflavones at 3, 27, 37, or 62 mg	9 weeks	Reduction in total and LDL-C levels by 4 and 6% with isolated soy protein with 62 mg of IFS vs casein. No change in plasma triglyceride and HDL-C concentrations.
Washburn et al., 1999 [90]	N = 51	Perimenopausal women (45–55 years)	Soy proteins (20 g) plus 34 mg of phytoestrogens (once daily or twice daily)	18 weeks	Significant declines in total cholesterol (6% lower) and LDL-C (7% lower). Significant decline in diastolic blood pressure (5 mm Hg lower) in the twice-daily soy diet, compared with the placebo diet. No significant effects for triglycerides, HDC-C or frequency of menopausal symptoms.
Merz.Demlow, 2000 [91]	N = 33	Premenopausal women (18–35 years)	Soy isoflavones (low: 64.7 ± 9.4 and high: 128.7 ± 15.7 mg/day)	Three menstrual cycles	Change in total cholesterol, HDL-C, and LDL-C concentrations across menstrual cycle phases (P < 0.005).
Takatsuka et al., 2000 [92]	N = 52	Premenopausal women (median age 26 years)	Soymilk (400 mL = 408 g) /day	Two menstrual cycles	Decrease of 10.9 mg/dL, or 5.3%, in serum total cholesterol concentrations in the soymilk-supplemented group.
Teede et al., 2001 [94]	N = 213	108 men and 105 post-menopausal women (50–75 years)	Soy proteins (40 g) and isoflavones at 118 mg/day	3 months	Reduction in the LDL-C/HDL-C ratio and triglyceride levels No change in total cholesterol, LDL-C and HDL-C concentrations.
Chiechi et, 2002 [93]	N = 187	Postmenopausal women (39–60 years)	Soy food every day with isoflavones amounting to 40–60 mg/day	6 months	Improved in lipid profile
Rosell et al., 2004 [95]	N = 1033	Pre-and postmenopausal women (≥20 years)	Soy proteins <0.5, 0.5–2.9, 3.0–5.9, and ≥6.0 g/day	n.a.	Inversely associated relationship between soy protein intake and total cholesterol and LDL-C concentrations and the ratio of total cholesterol to HDL-C, but not to HDL-C concentrations.
Derosa et al., 2018 [97]	N = 124	Men and women (≥18 years)	Fermented soybean extract	n.a.	Decreased of total cholesterol, TG and LDL-C levels. No change in HDL-C levels.
George et al., 2020 [98]	N = 135	65 men and 70 women (27–87 years)	Soy proteins 40 g/day	3 months	No effect on total cholesterol, HDL-C, or LDL-C levels. Reduction in bone alkaline phosphatase and body fat percentages.
Haddad Tabrizi, 2020 [99]	N = 31	12 men and 19 women (median age 61 years)	Lunasin enriched soybean extract 335 mg/day	8 weeks	No significant changes in serum lipids, glucose, insulin resistance, blood pressure, BMI, or waist circumference.

Abbreviations: HDL-C, high-density lipoprotein-cholesterol; LDL-C, low-density lipoprotein-cholesterol; n.a., not available; apo, apolipoprotein.
